# Gait Indicators Contribute to Screening Cognitive Impairment: A Single- and Dual-Task Gait Study

**DOI:** 10.3390/brainsci13010154

**Published:** 2023-01-16

**Authors:** Xiaoqin Wang, Wuhan Yu, Lihong Huang, Mengyu Yan, Wenbo Zhang, Jiaqi Song, Xintong Liu, Weihua Yu, Yang Lü

**Affiliations:** 1 Department of Geriatrics, The First Affiliated Hospital of Chongqing Medical University, Chongqing 400016, China; 2 Institutes of Neuroscience, Chongqing Medical University, Chongqing 400016, China

**Keywords:** cognitive impairment, gait parameters, toe-off ground angle, timed “Up & Go” test

## Abstract

Background: Screening cognitive impairment is complex and not an appliance for early screening. Gait performance is strongly associated with cognitive impairment. Objectives: *We aimed to explore gait indicators that could potentially screen cognitive dysfunction*. Methods: A total of 235 subjects were recruited from June 2021 to June 2022. Four gait tasks, including the walking test, the timed “Up & Go” test (TUG), foot pressure balance (FPB), and one-legged standing with eyes closed test (OLS-EC), were performed. Moreover, in the walking test, participants were instructed to walk at their usual pace for the single-gait test. For the dual-task tests, participants walked at their usual pace while counting backward from 100 by 1s. The data were analyzed by the independent sample *t*-test, univariate and multivariate logistic regression, a linear trend, stratified and interaction analysis, the receiver operating characteristic (ROC) curve, and Pearson’s correlations. Results: Among the 235 participants, 81 (34.5%) were men and 154 (65.5%) were women. The mean age of participants was 72 ± 7.836 years. The control, MCI, mild AD, and severe AD groups had means of 71, 63, 71, and 30, respectively. After adjusting for age, sex, education, and body mass index (BMI), the dual-task toe-off-ground angle (TOA) (odds ratio (OR) = 0.911, 95% confidence interval (CI): 0.847, 0.979), single-task TOA (OR = 0.904, 95% CI: 0.841–0.971), and the timed “Up & Go” time (TUGT) (OR = 1.515, 95% CI: 1.243–1.846) were significantly associated with an increased risk of cognitive impairment. In addition, the trend test and stratified analysis results had no significant differences (all *p* > 0.05). The area under the roc curve (AUC) values of TOA in the dual-task and TUGT were 0.812 and 0.847, respectively. Additionally, TOA < 36.75° in the dual-task, TOA < 38.90° in the single-task, and TUGT > 9.83 seconds (s) are likely to indicate cognitive impairment. The cognitive assessment scale scores were significantly correlated with TOA (all r > 0.3, *p* < 0.001) and TUGT (all r > 0.2), respectively. Conclusion: TOA and TUGT scores are, in some circumstances, associated with cognitive impairment; therefore, they can be used as simple initial screenings to identify patients at risk.

## 1. Introduction

Cognitive impairment is a common syndrome that encompasses a spectrum varying from mild cognitive impairment (MCI) to dementia, and MCI is considered to be an intermediate condition between “normal aging” and dementia [1]. MCI signifies a decline in cognitive function beyond typical aging without impacting functional activities [2]. The annual conversion rate to dementia is approximately 10% to 15% [3]. Dementia is a leading cause of disability and dependence in older adults, which affects their physical and mental health and places an economic and caregiving burden on society [4]. At the same time, the global prevalence of dementia is increasing as the proportion of older adults in the population increases [5]. To our knowledge, Alzheimer’s disease (AD) accounts for 60–70% of all dementia disorder cases and has become one of the deadliest diseases worldwide [6,7]. Therefore, the identification of MCI and early AD is needed to enable pharmacological treatment to slow the progression of dementia [8,9,10].

The clinic diagnosis of cognitive impairment may be extensive, involving medical history, physical examination, neuropsychological tests, blood tests, and neuroimaging [11]. However, the above methods are invasive, time-consuming, expensive, and not applicable for early screening. At the same time, some cognitive screening tools have some shortcomings, for example, Mini-Mental State Examination (MMSE) may miss patients in the early stage of cognitive impairment [12]. Moreover, most screening tools depending on language will be affected by education and age [13]. Therefore, noninvasive, less time-consuming, and low-cost methods to enhance early cognitive impairment screening have been called for [14].

Gait, as a behavioral characteristic of human walking, requires the cooperation of multiple systems, including the brain, nerves, bones, muscles and joints [15]. A safe and effective gait is closely related to the human health process across the life span [16] and is a predictor for cognitive decline [17,18,19]. Due to their robust clinical measurement characteristics, gait speed and stride length are widely used to evaluate, identify, and predict cognitive-related diseases [20,21,22]. However, gait speed [23] or stride length [24] are nonspecific variables that affect sensitivity and specificity if cognitive impairment is evaluated solely. Moreover, gait parameters are multidimensional [15]; one gait index thus cannot be used to summarize the characteristics of all diseases. Notably, recent studies have found that relevant indicators of gait rhythm, such as time domain (TD) and time-dependent spectral features (PSDTD), also provide advanced clinical method support for the identification of neurodegenerative diseases (NDD) [25]. Therefore, there is an urgent need to explore gait indicators for evaluating patients with cognitive impairment from multiple perspectives.

Recent studies have shown that cognitive impairment may be essential for elderly individuals suffering from abnormal balance [26,27]. The worsening of balance function parallels the severity of cognitive impairment. Therefore, we hypothesize that some representative indicators of the balance function test can be used to distinguish and screen cognitive impairment. As far as we know, the balance tests include the timed “Up & Go” test (TUG) [28], one-legged standing with eyes closed test (OLS-EC) [29] and foot pressure balance test (FPB) [30]. Among them, the TUG is one of the most useful evaluation methods in the health care field, because it is temporally and spatially efficient and contains various motion elements [28]. In addition, a recent study has confirmed the accuracy of this measurement method using an algorithm for calculating TUG phase and quantitative parameters by identifying leg movement and muscle contraction using an inertial and an electromyography sensor [28]. Similarly, the OLS-EC is suitable for large-scale population screening due to its easy operation and high sensitivity. The FPB, which measures the pressure distribution between the plantar and the supporting surface, can facilitate researchers’ study of the problems of balance, falling, and gait dysfunction from various angles and is an ideal tool for evaluating the current balance ability. At the same time, an increasing number of studies have adopted the dual-task gait paradigm, which requires subjects to walk while completing additional cognitive tasks [31]. Patients with cognitive impairment have an insufficient cognitive reserve and impaired ability to allocate attention and executive and visuospatial function in the dual-task paradigm, which can be used to more sensitively reflect pathological gait features [16,32]. Therefore, we have included single- and dual-task gait tests in walking to find new indicators to distinguish cognitive impairment.

In our present study, the walking and balance tests were combined to describe the gait characteristics of patients with cognitive impairment and normal cognition. At the same time, correlation analysis was performed between neuropsychological scales and the gait indicators. We hope it provides vital support for screening and diagnosing cognitive impairment.

## 2. Methods

This study was a cross-sectional study. Patients were recruited from the Memory Clinic, Department of Geriatrics, The First Affiliated Hospital of Chongqing Medical University, from June 2021 to April 2022. The study protocol was approved by the Medical Ethics Committee of The First Affiliated Hospital of Chongqing Medical University (approval number: 20212901; time of ethics approval: 10 May 2021).

### 2.1. Study Subjects

Inclusion criteria included the following: (1) 60 years and older; (2) ability to walk back and forth for 8 m independently; and (3) agreement to participate in this study. The exclusion criteria of all subjects included the following: (1) present in the stressed state; (2) comorbid severe cardiopulmonary diseases such as heart failure, myocardial infarction, or emphysema; (3) motor system dysfunction (such as osteoarthritis or knee/hip joint disease); (4) using neuropsychiatric drugs; and (5) refusal to participate in this study. First, the participants were divided into control, MCI, mild AD, and moderate AD groups. Next, we divided them into cognitive normal (control group) and impairment groups to perform regression modelling.

### 2.2. Data Collection 

Information about age, sex, height, weight, and education was recorded. Participants were divided into two age groups: young older (<70 years) and old older (>70 years) [33]. Education status was classified as higher (>9 years of education) and lower (1–9 years of education) [34]. Participants were also divided into four groups based on body mass index (BMI) according to the World Health Organization Asian adult bodyweight standard: obese (≥25.0 kg/m^2^), overweight (23.0–24.9 kg/m^2^), regular (18.5–22.9 kg/m^2^), and underweight (<18.5 kg/m^2^).

### 2.3. Cognitive Assessment 

Patients received neuropsychological assessments in face-to-face interviews. The assessments were performed by a trained nurse or an experienced doctor. The scales in our study were as follows: Chinese version of MMSE, Clock-Drawing Test (CDT), Trail Making Tests A (TMT A), Trail Making Tests B (TMT B), attention using the Digit Span Test forward (DSF) and Digit Span Test backward (DSB), Instrumental Activities of Daily Living (IADL), Geriatric Depression Scale (GDS), and Clinical Dementia Rating Scale (CDR).

### 2.4. Diagnosis

The diagnosis of cognitive impairment was made by experts in the memory clinic of The First Affiliated Hospital of Chongqing Medical University, combined with the above neuropsychological tests, medical history, physical examination, blood tests, and brain magnetic resonance imaging (MRI) or Computed Tomography (CT).

### 2.5. Gait Assessment

Before the trials, participants were given standardized instructions and a visual demonstration. Then, participants walked back and forth twice at a comfortable pace for 16 m under single- and dual-tasks while wearing comfortable shoes with sensor devices (GAIT Rite, version 4.5; CIR systems Inc., Dalian, China). Gait data were recorded and stored in a computer. Meanwhile, all data were complete and met the analysis requirements. The detailed test steps are as follows. In the walking test, for the single-gait test, participants were instructed to walk at their usual pace in a quiet environment, without using any mobility aids. For the dual-task tests, participants walked at their usual pace while counting backward from 100 by 1 s aloud. The rationale for the dual-task condition selection has been described elsewhere [35,36]. Additionally, only one trial was performed in each condition, while the order of the single- and dual-tasks was randomized to balance and minimize the effects of learning and tiredness. 

The specific operations of the balance tests were as follows. For the TUG, participants were asked to perform [37,38] (1) sitting quietly on a stool; (2) standing up from the stool while hearing “start”; (3) walking 3 m, turning, and walking back to the stool; and (4) sitting on the stool again. Similarly, for the OLS-EC [29], participants were told to raise one foot to the mid-calf level of the supporting leg while spreading their arms. The test stopped when the subject lowered the raised foot (i.e., connected it to the floor). The computer recorded the standing time, and the longest time was selected for each leg. For the FPB [30], participants were asked to stand in a static position with both legs for 20 s. The whole process took approximately 10 min. 

### 2.6. Statistical Analysis

The quantitative data were assessed as follows. The distributions of continuous measurements were presented by the mean and standard deviation (SD) (mean ± SD), whereas categorical variables were expressed as count and frequency (%). The independent samples *t*-test was used to analyze differences in variables. A binary logistic regression was used to evaluate the relationship between gait indicators and the risk of cognitive impairment. We performed a linear trend and stratified and interaction analysis to detect any dose-response association between gait parameters and cognitive impairment. The receiver operating characteristic (ROC) curve was assessed to evaluate the ability of gait indicators to differentiate disease. Pearson’s correlations were used to explore the gait indicators and cognitive scale correlations. Differences were considered significant at *p* < 0.05. Odds ratios (ORs) with 95% confidence intervals (CIs) were calculated. All statistical analyses were carried out by SPSS 26.0 (IBM, Armonk, NY, USA). Gradprism8 drew all figures.

## 3. Results

### 3.1. Baseline Characteristics of Participants Stratified by Disease Diagnosis

Among the 235 participants, 81 (34.5%) were men and 154 (65.5%) were women. The mean age of participants was 72 ± 7.836 years. The numbers of the control, MCI, mild AD, and severe AD groups were 71, 63, 71, and 30, respectively. Participant characteristics according to cognitive impairment status are shown in Table 1. Compared to the control group, both mild AD and moderate AD groups were older; The moderate AD group had lower education (*p* < 0.05). Compared to the control, both the MCI and mild AD groups had higher systolic BP (*p* < 0.05). Compared to the control, the mild cognitive impairment, mild AD, and moderate AD groups performed worse on the MMSE and CDT (*p* < 0.05).

### 3.2. Gait Characteristics in Different Severities of Cognitive Impairment

The gait characteristics of the control, the MCI, and the mild and moderate AD groups are presented in Table 2. Compared to the control group, the other three groups had a lower speed, a shorter stride length, longer stride time, decreased cadence, less braking force, smaller toe-off ground angle (TOA), smaller heel-to-ground angle (HOA), higher percentages in the standing phase, and lower percentages in the swing phase in single- or dual-task tests, respectively (*p* < 0.001). Additionally, the phenomenon of the gait characteristics’ decline is proportional to the severity of the cognitive impairment. Furthermore, compared to the control group, patients with MCI, mild AD, and moderate AD took longer times in the TUG (*p* < 0.001) and shorter times in the OLS-EC (*p* = 0.001), respectively. There was no difference in the FPB (total center *p* = 0.346, left foot *p* = 0.212, and right foot *p* = 0.98) among these groups. 

### 3.3. Association between the Timed “Up & Go” Time (TUGT), TOA, and Cognitive Impairment 

**Figure 1:**
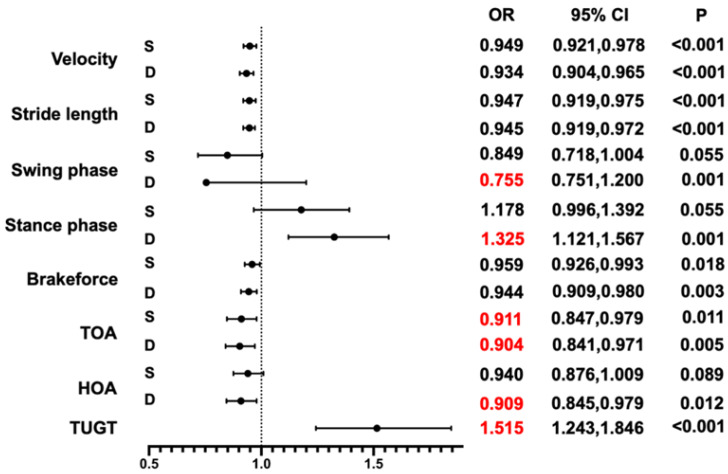
Forest map of multivariate logistic regression analysis for the association between gait indicators and a risk of cognitive impairment, where red represents significant associations both in dual- and single-task. Abbreviations: OR, odds ratio; CI, confidence interval. S, single-task; D, dual-tasks; TOA, toe-off ground angle; HOA, heel-to-ground angle; TUGT, timed “Up & Go” time.

The logistic regression model was used to analyze the associations between each gait index and cognitive impairment (Table 3). After adjusting for age, gender, education, and BMI, the gait speed, stride length, and TOA in single- and dual-tasks remained significantly different and so did TUGT (all *p* < 0.05). A forest map was drawn based on the results of the multiple logistic regression of model 3 (Figure 1). The ORs of the TOA tertiles 2 and 3 in a single-task for a risk of cognitive impairment were 0.217 [95% CI (0.079–0.592)] and 0.097 [95% CI (0.035–0.269)], respectively (*p* trend < 0.001). The ORs of the TOA tertiles 2 and 3 in dual-task for a risk of cognitive impairment were 0.174 [95% CI (0.061–0.497)] and 0.068 [95% CI (0.023–0.197)], respectively (*p* trend < 0.001). The ORs of the TUGT tertiles 2 and 3 for a risk of cognitive impairment were 11.966 [95% CI (4.937–29.005)] and 36.713 [95% CI (11.549–116.708)], respectively (*p* trend < 0.001) (Table 4). In addition, the stratified analysis by age, sex, education level, and BMI demonstrated that different populations did not affect the association between TOA, TUGT, and an increased risk of cognitive impairment (*p* > 0.05, Table 5).

**Table 2:** Comparison of multiple models of gait data in different individuals with cognitive impairment.

		Total	Control	MCI	Mild AD	Moderate AD	*p*-Value
Velocity, mean (SD), (cm/s)	S	75.13 (17.836)	87.30 (14.721)	76.33 (15.572)	69.46 (14.625)	56.83 (14.620)	<0.001 *
	D	64.01 (18.114)	78.68 (15.099)	64.28 (14.393)	55.29 (12.808)	43.58 (11.321)	<0.001 *
Stride length, mean (SD), (cm/s)	S	93.12 (18.769)	105.63 (13.137)	93.94 (17.652)	86.70 (16.840)	76.60 (17.470)	<0.001 *
	D	89.51 (21.331)	105.14 (14.069)	90.40 (18.884)	80.17 (19.362)	66.33 (14.445)	<0.001 *
Stride time (ms)	S	1.264 (0.143)	1.215 (0.135)	1.251 (0.134)	1.281 (0.131)	1.364 (0.159)	<0.001 *
	D	1.432 (0.209)	1.355 (0.208)	1.425 (0.218)	1.480 (0.188)	1.545 (0.155)	<0.001 *
Cadence (steps/min)	S	96.601 (10.154)	100.157 (10.304)	97.566 (9.446)	95.237 (9.061)	89.299 (9.627)	<0.001 *
	D	85.476 (11.354)	90.007 (11.555)	86.029 (11.679)	82.486 (9.885)	78.785 (7.953)	0.003 *
Swing phase (%)	S	32.860 (2.748)	34.368 (2.325)	33.381 (2.460)	32.025 (2.307)	30.120 (2.524)	<0.001 *
	D	31.116 (3.078)	33.166 (2.314)	31.358 (2.937)	29.235 (2.497)	27.867 (2.411)	<0.001 *
Stance phase (%)	S	67.140 (2.748)	65.632 (2.325)	66.619 (2.460)	67.975 (2.307)	69.880 (2.524)	<0.001 *
	D	68.884 (3.078)	66.834 (2.314)	68.642 (2.937)	70.146 (2.497)	72.133 (2.411)	<0.001 *
Back-force	S	0.687 (0.153)	0.759 (0.104)	0.707 (0.160)	0.648 (0.145)	0.563 (0.158)	<0.001 *
	D	0.661 (0.151)	0.755 (0.104)	0.677 (0.145)	0.599 (0.140)	0.514 (0.118)	<0.001 *
TOA	S	37.550 (7.102)	41.738 (4.934)	38.365 (7.001)	35.426 (6.489)	30.810 (6.253)	<0.001 *
	D	35.786 (7.571)	40.869 (5.268)	36.748 (7.119)	32.442 (6.519)	27.400 (5.085)	<0.001 *
HOA	S	24.510 (6.713)	28.018 (5.068)	25.671 (6.845)	22.286 (6.150)	18.883 (5.667)	<0.001 *
	D	23.252 (7.107)	27.876 (5.032)	24.350 (6.828)	19.852 (6.217)	16.038 (4.544)	<0.001 *
TUGT		12.830 (5.289)	9.588 (2.539)	12.339 (3.396)	14.388 (5.227)	19.927 (7.070)	<0.001 *
OLS-EC	L	4.03 (2.798)	4.26 (2.207)	4.78 (3.589)	3.56 (2.454)	2.68 (5.477)	0.001 *
	R	4.06 (2.986)	4.72 (2.617)	4.28 (3.796)	3.56 (2.628)	2.88 (2.007)	0.001 *
FPB	T	149.039 (96.785)	149.539 (93.665)	152.436 (107.737)	158.347 (93.728)	119.113 (84.543)	0.346
	L	141.016 (71.117)	140.374 (71.854)	149.691 (65.113)	136.331 (78.519)	134.430 (65.694)	0.212
	R	74.538 (131.111)	70.606 (128.401)	77.260 (137.101)	86.714 (140.232)	50.606 (102.710)	0.980

Data of continuous variables described as means ± standard deviation (SD). Abbreviations: MCI, mild cognitive impairment; AD, Alzheimer’s disease; TOA, toe-off ground angle; HOA, heel-to-ground angle; TUGT, timed “Up & Go” time; S, single-task; D, dual-tasks, counting backward 100 by 1s; OLS-EC: one-legged standing with eyes closed; FPB, foot pressure balance test; L, left foot; R, right foot; T, total gravity distribution; * *p* < 0.01 represents statistical significance.

### 3.4. Accuracy of TOA and TUGT for Predicting Patients with Cognitive Impairment

**Figure 2:**
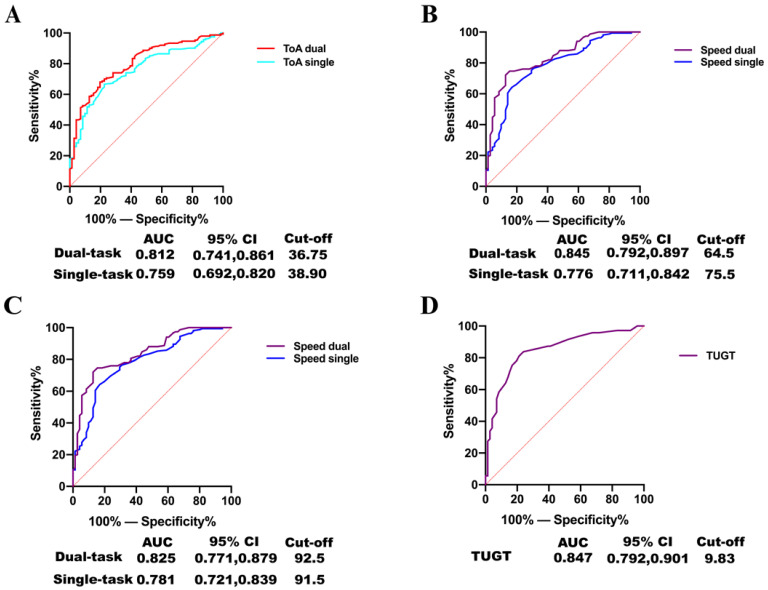
Gait indicators classifiers for cognitive normality and impaired diagnosis. ROC curves show the independent accuracy of gait indicators classifier disease diagnosis: ROC curve for TOA (**A**), speed (**B**), stride length (**C**), and TUGT (**D**), respectively. Abbreviations: ROC, receiver operating characteristic; OR, odds ratio; CI, confidence interval; AUC, area under the roc curve; TOA, toe-off ground angle; TUGT, timed “Up & Go” time.

TUGT, TOA, gait speed, and stride length were selected for ROC analysis according to the logistic regression results. The area under the ROC curve (AUC) of TUGT was 0.847. The AUCs of TOA, gait speed, and stride length in the dual-task were 0.812, 0.845, and 0.825, respectively. The AUCs of TOA, gait speed, and stride length in a single-task were 0.759, 0.776, and 0.781, respectively (Figure 2). In addition, the cut-off values of TOA in dual-task, TOA in single-task, and TUGT were 36.75°, 38.90°, and 9.83 s, respectively.

### 3.5. Association between TOA, TUGT, and Cognitive Assessment Scales

**Figure 3:**
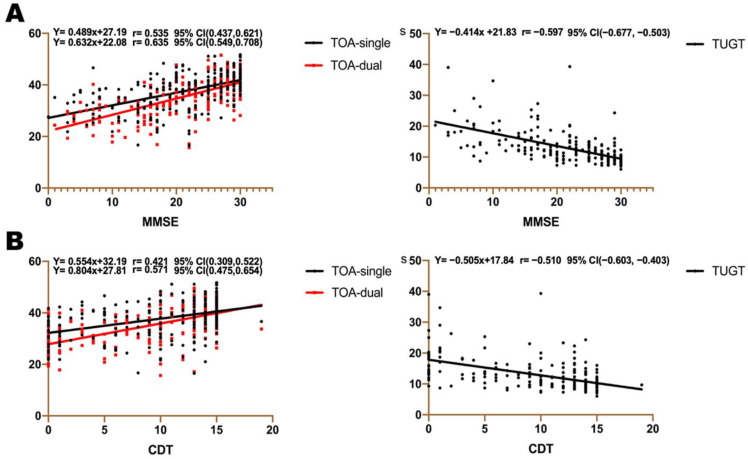

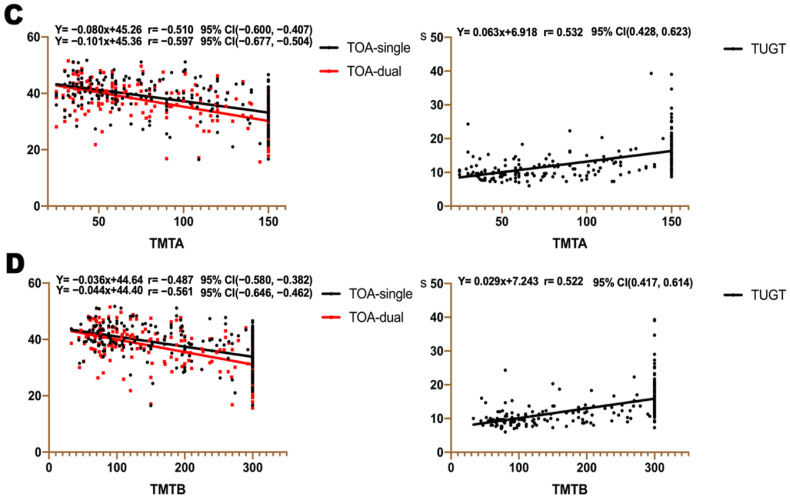

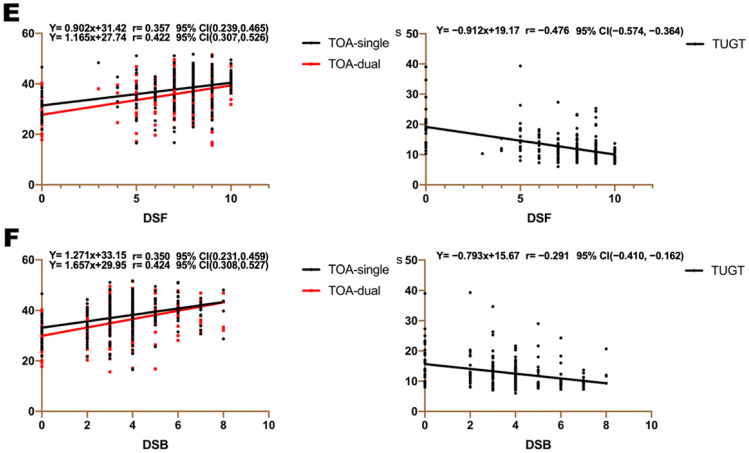
Correlations between gait indications and cognitive scales. Correlations between TOA, TUGT and (**A**) MMSE, (**B**) CDT, (**C**) TMT A, (**D**) TMT B; (**E**) DSF; (**F**) DSB; r = correlation coefficient. Abbreviations: MMSE, Mini-Mental State Examination; CDT, Clock-Drawing Test; TMT A, Trail Making Test A; TMT B, Trail Making Test B; DSF, Digit Span Test forward; DSB, Digit Span Test backward; TOA, toe-off ground angle; TUGT, timed “Up & Go” time.

The Pearson’s correlation was used to reflect the association between TOA, TUGT, and the cognitive assessment scales (Figure 3). A line was then fit by linear regression and showed that the correlations between single-task TOA and MMSE, CDT, TMTA, and TMTB were 0.535, 0.421, 0.510, and −0.487, respectively. The correlations between dual-task TOA and MMSE, CDT, TMTA, and TMTB were 0.635, 0.571, 0.597, and −0.561, respectively. The correlations between TUGT and MMSE, CDT, TMTA, and TMTB were −0.597, −0.510, 0.532, and 0.522, respectively.

## 4. Discussion

### 4.1. Our Findings in This Study

The present study indicates that TOA and TUGT are independent and stable factors for the risk of cognitive impairment. Gait indicators such as TOA and TUGT become more obvious abnormalities with the exacerbation of the cognitive impairment. Additionally, TOA < 36.75° in the dual-task, TOA < 38.90° in the single-task, and TUGT > 9.83 s are likely to indicate cognitive impairment with moderate sensitivity and accuracy. Moreover, the TOA and TUGT show a strong correlation with cognitive domains. The gait paradigm we adopted can distinguish control, MCI, mild AD, and moderate AD. Gait indicators in dual-task conditions are more sensitive than single-task indicators in determining healthy people’s cognitive impairment. 

### 4.2. TOA and TUGT Are Unique and New Gait Indexes

TOA and TUGT are independent and stable factors of cognitive impairment risk. To our knowledge, this result has not been mentioned in previous studies. TOA belongs to the posture-control domains. Indeed, the posture data have many descriptors, such as temporal [39], geometrical [40], and non-linear parameters [41], which have proved to be sensitive to evaluating the postural steadiness. Most studies require the model transformation to deal with the data before assessing postural stability, whereas our research can use the TOA and TUGT directly for an assessment. In addition, from a kinematic analysis, the postural-control disorder in the elderly may reflect subclinical pathologies affecting one or more components of the postural-control system and age-related changes in the sensorimotor systems. Most patients with cognitive impairment have pathological brain changes and information introduction disorders of visual, vestibular, and somatosensory systems [39]. Thus, they will accrue complex sensorimotor dysfunction and finally show posture changes. From a pathological point of view, patients with cognitive impairment have visuospatial deficits due to occipital atrophy and local neurodegeneration [42]. Visuospatial impairment can affect postural control. Additionally, if the prefrontal cortex is damaged, executive abnormalities will occur, leading to abnormal posture presentations [42,43]. Previous studies show that many cognitively impaired patients have motor and pathological system dysfunctions, so posture-related measurements will change accordingly [43]. Additionally, in our study, we found a significant difference in TOA between the control, MCI, and AD populations, which also supports the view that TOA belongs to the field of posture domain. Furthermore, the trend test and stratified analysis results confirm that individual differences do not influence TOA, TUGT, and the risk of cognitive impairment, which supports our viewpoint. 

### 4.3. Cognitive Impairment and Gait Impairment Have a Strong Association

The severity of cognitive impairment is positively related to gait impairment. Our results illustrate that decreased TOA and increased TUGT scores are negatively correlated with scores on cognitive assessment scales. Gait indicators, including TOA and TUGT, could thus be progressive markers for cognitive impairment. It is likely that there is a decreased volume of gray matter in the cerebral cortex, basal ganglia, and caudate nucleus in patients with cognitive impairment [44,45]. Additionally, TOA and TUGT are associated with the parietal, frontal, and internal olfactory cortices, which affect overall cognitive, visuospatial, attention, and executive function, respectively [46,47,48,49,50]. This result also demonstrates that TOA and TUGT change as cognitive severity varies. The TOA and TUGT have the capability to predict the severity of cognitive impairment. 

### 4.4. Balance Function Tests (TUG and the OLS-EC) Are Useful for Cognitive Impairment Patients

It is known that the balance function has two states, including static and dynamic balance [51]. The TUG and OLS-EC tests belong to dynamic balance, while the FPB test is static equilibrium. The body moves while generating corresponding internal disturbances, leading to an increased brain burden, and greater susceptibility to balance dysfunction [52,53]. Our results indicate that the TUG and the OLS-EC tests show significant differences among the four groups except for the FPB test. This phenomenon confirms that patients with MCI and AD have impaired dynamic balance. 

### 4.5. The Gait Paradigms Can Successfully Detect Different Severities of Cognitive Impairment Patients

The gait paradigms we adopted can successfully detect control, MCI, mild AD, and moderate AD. In the walking test, single- and dual-tasks would involve two simultaneously performed tasks that would interfere and compete for cortical brain resources [54]. Consequently, we chose the dual-task (counting from one hundred to one) [36] based on an established cognitive function test consistent with participants’ ability thresholds [55] without causing undue stress [56]. Furthermore, the TUG, OLS-EC, and FPB are all ideal tools for assessing balance [30,37,57]. The gait paradigms showed significant variability across the four groups. Therefore, the gait paradigms are applied to screen the present study population.

### 4.6. Gait Indicators Are More Sensitive in Dual-Task Than Single-Task Assessments

Gait indicators are more sensitive in dual-task than single-task assessments to differentiate between patients with cognitive impairment and normal cognition. It could be possible that walking requires more attention during a dual-task test [31]. The dual-task gait test is unique because it reflects the motor–cognitive interface. Cognitive and motor controls share common brain networks, which may become more overloaded when motor tasks are performed alongside cognitive tasks, particularly in people with cognitive impairment who have a lower cognitive reserve [32,56,58]. Moreover, the cut-off value shows that TOA is minor under dual-task conditions, unlike single-task conditions, implying that dual-task states can distinguish cognitive impairment patients within a slight window. This strongly supports our view. 

### 4.7. Gait Facilitates the Clinical Assessment of Patients with Cognitive Impairment

A comprehensive neuropsychological test usually takes more than 2 h. In contrast, our process only takes approximately 10 min, and the test effectively saves time. In addition, the clinical diagnosis of AD often requires cognitive assessments, cranial MRI, and blood tests, as well as biomarkers from positron emission tomography (PET) and cerebrospinal fluid (CSF), such as Aβ42, total tau, and phosphorylated-tau [59]. However, cranial MRI, PET, and genetic tests are costly, and CSF tests are more traumatic for the patient. CSF, MRI, and PET are impractical, costly, and invasive, as well as not being suitable for large-scale early screening, whereas gait is not only low-cost and non-invasive, but also saves examination time and is ideal for early screening for diseases. Therefore, the gait test adopted in this study is easy for clinical application. Similarly, gait indicators have the potential to be simple initial screenings to identify patients at risk.

### 4.8. Strengths

Our study has several strengths. Firstly, we screen out new indicators which can be used as an index to screen for cognitive impairment through four gait paradigms and to prove their independence and stability. Secondly, we explore the strong correlation between TOA, TUGT, and cognitive scales. Thirdly, our outcomes are easily transformed into clinical application because of the simplicity, noninvasiveness, and low cost of gait manipulation. Finally, TOA and TUGT provide a reliable screening range for clinical applications.

### 4.9. Limitations

Our study has some limitations. First, our study design is cross-sectional, which makes it challenging to explain the causality between gait parameters and cognitive impairment. Second, although gait is simple to operate, non-invasive, and low cost, it is only suitable for patients without motor dysfunction. Therefore, the gait data of patients with dyskinesia are missing. Third, because the participants are enrolled from a single-center memory clinic in Chongqing, southwest China, our results do not represent the gait characteristics of the elderly in other areas. Therefore, future studies should cross-examine this approach in different cohorts of cognitive impairment to support its clinical applicability. 

## 5. Conclusions

This study showed that TOA and TUGT were independent and stable factors for the risk of cognitive impairment. TOA < 36.75 (dual-task), TOA < 38.90 (single-task), and TUGT > 9.83 seconds (s) may indicate cognitive dysfunction with moderate sensitivity and accuracy. In brief, TOA and TUGT scores are, in some circumstances, associated with cognitive impairment; therefore, they can be used as simple initial screenings to identify patients at risk.

## Figures and Tables

**Table 1:** Baseline characteristics of participants stratified by disease diagnosis.

Variable	Total	Control	MCI	Mild AD	Moderate AD	*p*-Value
	N = 235	N = 71	N = 63	N = 71	N = 30	
Age, mean SD	72.0 (7.836)	71.01 (6.737)	70.05 (7.985)	73.41 (7.989)	75.10 (8.372)	0.007 *
Female, N (%)	154 (65.5)	50 (70.4)	42 (66.7)	46 (64.8)	16 (53.3)	0.427
Height, mean (SD, (cm)	1.584 (0.080)	1.593 (0.0798)	1.581 (0.0827)	1.590 (0.0724)	1.583 (0.0905)	0.816
Weight, mean (SD, (Kg)	57.222 (9.872)	59 (9.368)	57.854 (10.634)	55.287 (9.428)	56.233 (8.721)	0.169
BMI, mean (SD, (kg/m²)	22.827 (3.526)	23.186 (2.740)	23.079 (3.429)	21.896 (3.562)	22.484 (3.215)	0.112
Education, mean (SD), (y)	9.3 (4.4)	11.817 (9.206)	8.817 (4.180)	9.282 (4.667)	6.10 (4.791)	<0.001 *
Cognitive tests						
MMSE, mean (SD)	21.23 (7.741)	28.82 (2.045)	23.33 (3.910)	17.31 (4.717)	8.10 (4.536)	<0.001 *
CDT score, mean (SD)	9.70 (5.387)	13.83 (2.813)	10.41 (4.272)	7.93 (4.894)	2.60 (3.936)	<0.001 *
DSF, mean (SD)	6.85 (2.790)	8.56 (1.105)	7.19 (1.608)	6.802.326)	2.03 (3.235)	<0.001 *
DSB, mean (SD)	3.48 (1.943)	5.06 (1.413)	3.53 (1.423)	2.97 (1.307)	0.76 (1.704)	<0.001 *
TMT A, mean (SD), s	96.72 (44.724)	51.97 (20.856)	99.69 (39.203)	117.29 (35.690)	147.00 (12.077)	<0.001 *
TMT B, mean (SD), s	199.48 (95.669)	98.59 (54.673)	210.74 (82.921)	247.31 (66.466)	300.00 (0)	<0.001 *
His, mean (SD)	2.64 (2.080)	1.87 (1.656)	2.86 (2.023)	2.97 (2.169)	3.28 (2.463)	<0.001 *
IADL, mean (SD)	12.51 (5.430)	8.62 (1.543)	10.49 (2.375)	14.04 (4.680)	22.37 (3.891)	<0.001 *
GDS, mean (SD)	5.62 (4.664)	3.69 (3.602)	7.02 (5.037)	6.20 (5.157)	5.88 (3.059)	<0.001 *

Data presented as mean ± standard deviation (SD) for continuous variables and percentage for dichotomous variables. Abbreviations: MCI, mild cognitive impairment; AD, Alzheimer’s disease; BMI, body mass index; MMSE, Mini-Mental State Examination; CDT, Clock-Drawing Test; DSF, Digit Span Test forward; DSB, Digit Span Test backward; TMT A, Trail Making Test A; TMT B, Trail Making Test B; IADL, Instrumental Activities of Daily Living; GDS, Geriatric Depression Scale; * *p* < 0.01 represents statistical significance.

**Table 3:** Univariate and multivariate logistic regression models evaluating the associations of gait indicators with cognitive impairment.

		Model 1			Model 2			Model 3		
		OR	95% CI	*p*-Value	OR	95% CI	*p*-Value	OR	95% CI	*p*-Value
**Gait variable (continuous)**										
Speed	S	0.952	0.928, 0.977	<0.001	0.936	0.884, 0.991	0.024	**0.949**	0.921, 0.978	<0.001
	D	0.935	0.908, 0.963	<0.001	0.928	0.899, 0.958	<0.001	**0.934**	0.904, 0.965	<0.001
Stride length	S	0.95	0.926, 0.975	<0.001	0.94	0.914, 0.967	<0.001	**0.947**	0.919, 0.975	<0.001
	D	0.947	0.924, 0.971	<0.001	0.94	0.915, 0.965	<0.001	**0.945**	0.919, 0.972	<0.001
Stride time	S	7.889	0.591, 105.305	0.118						
	D	4.78	0.871, 26.235	0.072						
Cadence	S	0.974	0.940, 1.008	0.134						
	D	0.971	0.942, 1.002	0.063						
Swing phase	S	0.839	0.723, 0.974	0.021	0.805	0.686, 0.945	0.008	0.849	0.718, 1.004	0.055
	D	0.754	0.644, 0.883	<0.001	0.727	0.614, 0.859	<0.001	0.755	0.751, 1.200	0.001
Stance phase	S	1.192	1.026, 1.384	0.021	1.242	1.058, 1.457	0.008	1.178	0.996, 1.392	0.055
	D	1.326	1.133, 1.552	<0.001	1.376	1.164, 1.628	<0.001	1.325	1.121, 1.567	0.001
Brakeforce	S	0.966	0.938, 0.994	0.016	0.949	0.917, 0.981	0.002	0.959	0.926, 0.993	0.018
	D	0.951	0.923, 0.980	0.001	0.935	0.902, 0.969	<0.001	0.944	0.909, 0.980	0.003
TOA	S	0.907	0.852, 0.967	0.003	0.890	0.831, 0.953	0.001	**0.911**	0.847, 0.979	0.011
	D	0.895	0.839, 0.954	0.001	0.885	0.827, 0.947	<0.001	**0.904**	0.841, 0.971	0.005
HOA	S	0.935	0.881, 0.993	0.028	0.916	0.857, 0.979	0.009	0.940	0.876, 1.009	0.089
	D	0.904	0.849, 0.963	0.002	0.890	0.831, 0.953	0.001	0.909	0.845, 0.979	0.012
TUGT		1.45	1.218, 1.727	<0.001	1.550	1.278, 1.879	<0.001	**1.515**	1.243, 1.846	<0.001
OLS-EC	L	1.072	0.952, 1.208	0.252						
	R	0.951	0.847, 1.069	0.403						

Model 1, no adjustment; model 2, adjusted for age and gender; model 3, adjusted for age, gender, education, BMI. Bold fonts represent the first five indicators more relevant to cognitive impairment. Abbreviations: TOA, toe-off ground angle; HOA, heel-to-ground angle; TUGT, timed “Up & Go” time; OLS-EC, one-legged standing with eyes closed; S, single-task; D, dual-tasks, counting backward 100 by one; L, left foot; R, right foot; T, total gravity distribution; OR, odds ratio; CI, confidence interval.

**Table 4:** Logistic regression analyses on associations among TOA, TUGT, and risk of cognitive impairment.

	Model 1 (Unadjusted)		Model 2		Model 3	
Variable	OR	95% CI	OR	95% CI	OR	95% CI
**Gait variable (Tertiles)**						
TOA (single-task)						
T1 < 35.244	1		1		1	
T2 35.244–40.732	0.187	0.071, 0.489	0.173	0.065, 0.461	0.217	0.079, 0.592
T3 > 40.732	0.078	0.031, 0.201	0.071	0.026, 0.191	0.097	0.035, 0.269
***p* trend**	<0.001 *		<0.001 *		<0.001 *	
TOA (dual-task)						
T1 < 32.5	1		1		1	
T2 32.5–39.952	0.154	0.055, 0.434	0.151	0.054, 0.428	0.174	0.061, 0.497
T3 > 39.952	0.054	0.020, 0.149	0.051	0.018, 0.146	0.068	0.023, 0.197
***p* trend**	<0.001 *		<0.001 *		<0.001 *	
TUGT						
T1 < 9.67	1		1		1	
T2 9.67–13.33	10.565	4.779, 23.358	12.381	5.269, 29.094	11.966	4.937, 29.005
T3 > 11.33	30.522	10.872, 85.686	41.262	13.393, 127.119	36.713	11.549, 116.708
***p* trend**	<0.001 *		<0.001 *		<0.001 *	

Gait variable was classed into tertiles; Abbreviations: OR, odds ratio; CI, confidence interval. TOA, Toe-off ground angle; TUGT, timed “Up & Go” time; * *p* for trend represents statistical significance.

**Table 5:** Stratified analysis and interaction between TOA, TUGT and risk of cognitive impairment.

Variable			TOA						TUGT		
	Single-Task					Dual-Task					
	Control	CIM	OR	95% CI	Interaction	OR	95% CI	Interaction	OR	95%CI	Interaction
					*p*-Value			*p*-Value			*p*-Value
**Total sample**	71 (30.2)	164 (69.8)		0.781, 0.891					1.62	1.351, 1.944	
**Age**											
Young older	28 (36.8)	48 (63.2)	0.884	0.811, 0.964	0.341	0.876	0.808, 0.949	0.158	1.712	1.247, 2.351	0.662
Old older	43 (27)	116 (73)	0.836	0.773, 0.903		0.806	0.741, 0.876		1.62	1.334, 1.968	
**Gender**											
Male	21 (25.9)	60 (74.1)	0.898	0.827, 0.975	0.141	0.822	0.738, 0.915	0.726	1.311	1.067, 1.612	0.022
Female	50 (32.5)	104 (67.5)	0.825	0.763, 0.892		0.852	0.796, 0.912		1.891	1.495, 2.394	
**Education**											
Lower	28 (21.7)	101 (78.3)	0.837	0.768, 0.912	0.375	0.797	0.721, 0.881	0.173	1.892	1.42, 2.521	0.147
Higher	43 (40.6)	63 (59.4)	0.882	0.816, 0.954		0.869	0.807, 0.935		1.461	1.198, 1.782	
**BMI**											
Underweight	3 (13.6)	19 (86.4)	0.866	0.705, 1.063	0.278	0.879	0.743, 1.04	0.099	NA		
Normal	28 (26.9)	76 (73.1)	0.824	0.747, 0.909		0.782	0.701, 0.872		1.614	1.267, 2.056	0.118
Overweight	24 (46.2)	28 (53.8)	0.852	0.764, 0.950		0.799	0.698, 0.915		1.786	1.243, 2.567	
Obese	18 (28.6)	45 (71.4)	0.894	0.808, 0.988		0.929	0.855, 1.01		1.358	1.041, 1.771	

Association between TOA, TUGT, and cognitive impairment status according to the Sub-group analysis of Age, Gender, Education, and BMI. Abbreviations: BMI, body mass index; CIM, cognitive impairment; CI, confidence interval; OR, odds ratio; TOA, toe-off ground angle; TUGT, timed “Up & Go” time.

## Data Availability

The data that support the findings of this study are available on request from the author Yang Lü. The data are not publicly available due to privacy and ethical restrictions.
